# Radiomics in Oncological PET Imaging: A Systematic Review—Part 1, Supradiaphragmatic Cancers

**DOI:** 10.3390/diagnostics12061329

**Published:** 2022-05-27

**Authors:** David Morland, Elizabeth Katherine Anna Triumbari, Luca Boldrini, Roberto Gatta, Daniele Pizzuto, Salvatore Annunziata

**Affiliations:** 1 Nuclear Medicine Unit, TracerGLab, Department of Radiology, Radiotherapy and Hematology, Fondazione Policlinico Universitario A. Gemelli, IRCCS, 00168 Rome, Italy; elizabethkatherineanna.triumbari@guest.policlinicogemelli.it (E.K.A.T.); danieleantonio.pizzuto@guest.policlinicogemelli.it (D.P.); salvatore.annunziata@policlinicogemelli.it (S.A.); 2 Service de Médecine Nucléaire, Institut Godinot, 51100 Reims, France; 3 Laboratoire de Biophysique, UFR de Médecine, Université de Reims Champagne-Ardenne, 51100 Reims, France; 4 CReSTIC (Centre de Recherche en Sciences et Technologies de l’Information et de la Communication), EA 3804, Université de Reims Champagne-Ardenne, 51100 Reims, France; 5 Radiotherapy Unit, Radiomics, Department of Radiology, Radiotherapy and Hematology, Fondazione Policlinico Universitario A. Gemelli, IRCCS, 00168 Rome, Italy; luca.boldrini@policlinicogemelli.it (L.B.); roberto.gatta.bs@gmail.com (R.G.); 6 Department of Clinical and Experimental Sciences, University of Brescia, 25121 Brescia, Italy; 7 Department of Oncology, Lausanne University Hospital, 1011 Lausanne, Switzerland

**Keywords:** radiomics, artificial intelligence, brain tumors, head and neck tumors, lung tumors, breast tumors, thyroid nodules, thymic tumors

## Abstract

Radiomics is an upcoming field in nuclear oncology, both promising and technically challenging. To summarize the already undertaken work on supradiaphragmatic neoplasia and assess its quality, we performed a literature search in the PubMed database up to 18 February 2022. Inclusion criteria were: studies based on human data; at least one specified tumor type; supradiaphragmatic malignancy; performing radiomics on PET imaging. Exclusion criteria were: studies only based on phantom or animal data; technical articles without a clinically oriented question; fewer than 30 patients in the training cohort. A review database containing PMID, year of publication, cancer type, and quality criteria (number of patients, retrospective or prospective nature, independent validation cohort) was constructed. A total of 220 studies met the inclusion criteria. Among them, 119 (54.1%) studies included more than 100 patients, 21 studies (9.5%) were based on prospectively acquired data, and 91 (41.4%) used an independent validation set. Most studies focused on prognostic and treatment response objectives. Because the textural parameters and methods employed are very different from one article to another, it is complicated to aggregate and compare articles. New contributions and radiomics guidelines tend to help improving quality of the reported studies over the years.

## 1. Introduction

The strive for personalized medicine, particularly in the oncological field, has led to the need to consider an ever-increasing amount of data to propose the most appropriate treatment at the most appropriate timing for each patient, so as to increase survival outcomes. Some recently arisen scientific fields are based on the measurement of biological molecules in a high-throughput way and attempt to comprehensively understand the underlying biology of systems of interest at the highest resolution possible [1]. The rationale of these so-called “omics” disciplines is to generate a reliable prognostic or predictive model for a certain condition, which should be more accurate than already existing models that were constructed based on conventionally collected clinical data [1]. The first “omic” disciplines were born in the late 1980s and were represented by genomics, proteomics, and metabolomics. Their importance was soon so well acknowledged that an “Omics era” was recognized existing from the end of the twentieth century. Taking advantage of the digital transformation in healthcare, medical imaging also contributed to the edifice by proposing its own “omic” field, that went under the name of “radiomics”. Radiomics corresponds to the extraction of a high number of quantitative features from medical images, beyond the dimensional, uptake, or volume parameters traditionally used in radiology and nuclear medicine. Their extraction is based on a rigorous processing chain where each step can greatly influence the result [2] and hinder the reproducibility of the findings. Interestingly, a recent study aimed to evaluate 77 oncology-related radiomics studies by proposing an objective measurement of radiomics research quality and concluded that the overall scientific quality and reporting of radiomics was insufficient [3]. It highlighted the frequent absence of a validation cohort and underlined that the most frequent limitations to reproducibility were represented by frequently missing data, insufficient reporting of study objectives, blind assessment, and sample size. Moreover, a major criticism was addressed to the low frequency in which demonstration of clinical utility was explained in those onco-radiomics articles.

The large amount of data generated by radiomics can be difficult to handle for traditional statistical approaches. Artificial intelligence (AI), with its ability to identify patterns within a massive dataset, is highly useful in this setting. This term covers several interrelated categories, including machine learning, which refers to all modeling and prediction applications based on training data (e.g., logistic regression), and deep learning, a sub-category of machine learning based on a neural network that is supposed to reproduce—on a smaller scale—the functioning of a human brain [4]. One of the key points of AI is the training base, which must be large enough to avoid overfitting issues [5], or, in other words, to avoid that the model becomes too attuned to the training data and loses its applicability to any other dataset.

As a result of the increasing number of articles on radiomics in oncological Positron Emission Tomography (PET) imaging, we here provide a systematic review of the literature, with a particular focus on assessing the quality of articles. In this first part, we will consider only supradiaphragmatic malignancies, other cancers will be treated in part 2.

## 2. Materials and Methods

This systematic review of published literature was performed according to the reporting standards of the PRISMA-P statement [6]. It was not registered.

### 2.1. Search Strategy, Inclusion and Exclusion Criteria

We performed a literature search in the PubMed database to identify all eligible articles using the following formula:

(“PET” OR “positron”) AND (“radiomics” OR “radiomic” OR “texture” OR “textural”)(1)

Results were admitted from 1 January 1990 up to and including 18 February 2022. Reviews were automatically identified using the article type options and removed from the extracted database.

Inclusion criteria were (Table 1): (1) studies based on human data, (2) studies specifying at least one supradiaphragmatic tumor type, and (3) studies performing radiomics on PET imaging. Exclusion criteria were: (1) studies not related to medical topics, (2) reviews, posters, editorials, comments, cases reports, (3) duplicates, (4) studies outside the oncological field or radiomics not performed on PET, (5) studies only based on phantom or animal data, (6) technical articles (optimization, robustness), without a clinically oriented question, (7) studies including fewer than 30 patients in the training cohort (for studies including multiple types of cancers, each cancer type was considered separately), (8) not strictly supradiaphragmatic malignancy (e.g., esophagus), (9) studies not in English, and (10) full text not available (Table 1).

### 2.2. Quality Assessment

Studies were assessed for quality based on three items:

1. The number of patients, estimating the risk of bias and overfitting: fewer than 50 patients (score 0), 50 to 100 patients (score 1), more than 100 patients (score 2);
2. The retrospective (score 0) or prospective (score 2) nature of the collection of data;
3. The use of a completely independent cohort for validation: no or k-folding—as it can expose to data leakage—(score 0), partition of the cohort between completely separated training and test set (score 1), external validation cohort (score 2).

A simple quality score (QS), consisting in the sum of the 3 previously stated items, was calculated. A maximum possible score of 6 meant high quality study design of the article. Mean and 95% confidence intervals (CI) of the quality scores were calculated for all database articles divided by year of publication.

Results from articles with a QS strictly lower than 3 were not considered in the result section.

### 2.3. Textural Parameters Used

The number of textural parameters that can be extracted in an image is huge. They can be grouped into several categories, which were assessed in this review [7,8]:

- Shape features: it is a purely geometric description of the segmented volume (metabolic tumoral volume, sphericity).
- First order features (also called histogram-based features): these parameters are based on the value of each voxel included in the segmented region without taking into account their spatial inter-relationships (maximum, minimum, average, standard deviation, etc.).
- Second order features: these parameters take into account the spatial interrelations between groups of pixels and are computed from texture matrices, calculated from the segmented region of interest [9]. Let us mention as an example Gray-level co-occurrence matrix (GLCM), which represents the frequency of occurrence of two intensity levels in neighboring voxels within a specific distance along a fixed direction or Gray-level run-length matrix (GLRLM), which encodes the size of homogenous runs for each image intensity.
- Higher order features: higher-order statistics features are computed after the application of specific mathematical transformations or filters [8].

### 2.4. Data Collection and Review

An Excel review database was generated. The database was filled in completely three times (two readings one week apart by one author and a second reading by a second one). Any discrepancies were corrected by consensus. The following parameters were extracted from each article:

- PMID, first author, year of publication;
- Organ/type of cancer;
- Quality data: number of patients, retrospective or prospective nature, validation, quality score;
- Objective of the study;
- Maximal order of textural parameter (shape only, first order, second order, higher order).

## 3. Results

### 3.1. Discrepancies between the Two Reading Sessions

Eleven discrepancies between the two reading sessions of the database were encountered and led to a third reading: one duplicate was identified, two articles were misclassified regarding cancer subtype, eight discrepancies concerned patient number and presence of a validation cohort.

### 3.2. Searching Results

**Figure 1:**
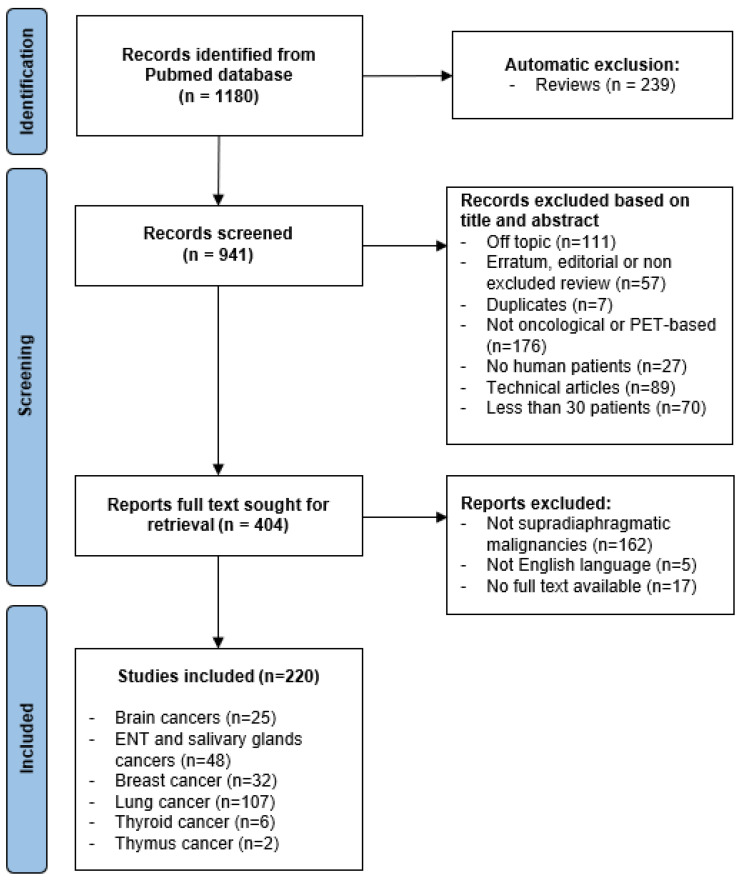
Flowchart of literature search and article selection.

A total of 1180 studies were identified in the PubMed database, 239 of which were reviews and therefore automatically excluded. Of the remaining 941 studies, 537 more were excluded as 111 were off topic, 57 articles corresponded to undetected reviews or editorials, 7 were duplicates, 176 articles were not oncological or based on PET-radiomics, 27 were not human-based, 89 were technical articles, and 70 included fewer than 30 patients in the training cohort. A total of 404 articles were then sought for retrieval: 5 were not written in English, 17 articles had no full text available, and 162 studies dealt with non-supradiaphragmatic malignancies and were therefore excluded. Finally, 220 studies were included in this review (Figure 1). A study characteristics table is available in a separate file (Supplementary Table S1).

### 3.3. Quality Assessments

Mean quality score of the articles was 2.05/6, with a constant improvement over the years (from 0.80 in 2014, to 1.96 in 2018, to 2.33 in 2021), as displayed in Table 2. A total of 119 (54.1%) studies included more than 100 patients each, 21 studies (9.5%) were based on prospectively acquired data, 91 (41.4%) articles described an independent validation set. The number of publications was found to increase each year (Table 2).

### 3.4. Textural Parameters Used

The vast majority of the included studies (n = 189, 85.9%) used both first and second-order textural parameters. Eight studies (3.6%) used only first-order parameters. Finally, 23 studies (10.5%) used higher order textural parameters.

### 3.5. Brain Cancers

A total of 23 of the 220 studies focused on oligodendrogliomas, gliomas, and glioblastomas [10,11,12,13,14,15,16,17,18,19,20,21,22,23,24,25,26,27,28,29,30,31,32] employing several PET tracers: 11C-Metionine (n = 7), 18F-FET (n = 7), 18F-FDG (n = 4), 18F-FDOPA (n = 3), 18F-FLT (n = 1), 18F-FMISO (n = 1). These studies included an average of 78.4 patients (range: 32–160), 6/23 (26.1%) including more than 100 patients, 3/23 (13.0%) with prospective data and 8/23 (34.8%) with validation sets. Main subjects included mutations and phenotyping (9/23, 39.1%), prognosis assessment (7/23, 30.4%), and differentiation between progression and radionecrosis (4/23, 17.4%).

In particular, three studies describing more than 100 patients used radiomics to predict O6-methylguanine-DNA methyltransferase promoter methylation status [12], isocitrate dehydrogenase phenotype [15], and mutations in the telomerase reverse transcriptase promoter status [28], with encouraging results. As for differentiation between progression and radionecrosis, studies showed heterogeneous results both in terms of tracer and performance. Wang et al. [22] reported promising results with 11C-Metionine (160 patients—112 of which in the training cohort, AUC of 0.914), whereas Ahrari [31] noted a poor added value of PET radiomics using 18F-FDOPA PET in patients with high-grade glioma.

Finally, 2 additional studies [33,34] focused on brain metastases, QS was however low.

### 3.6. Head and Neck Cancer including Salivary Gland Cancers

A total of 48 articles met the inclusion criteria in the group of head and neck malignancies [35,36,37,38,39,40,41,42,43,44,45,46,47,48,49,50,51,52,53,54,55,56,57,58,59,60,61,62,63,64,65,66,67,68,69,70,71,72,73,74,75,76,77,78,79,80,81,82], 46/48 (95.8%) using 18F-FDG. Only 1 study out of 48 (2.1%) used 18F-FMISO [36] and 1/48 (2.1%) [42] used 18F-FLT. However, the conclusions of these two articles remain limited due to the small number of patients studied (between 30 and 35). Included articles had an average of 155.5 patients (range 30–707) with 24/48 (50.0%) studies including more than 100 patients and 24/48 (50.0%) using an independent validation cohort.

A vast majority of these articles (37/48, 77.1%) focused on prognostic issues by offering various and heterogeneous models. In a vast retrospective study on patients with nasopharyngeal carcinoma (470 patients in the training set and 237 in the test set), Peng et al. [61] used a deep-learning approach to predict disease-free survival with a C-index of 0.754 (95%CI: 0.709–0.800) and potentially guide the induction chemotherapy. Notably, the only negative study was conducted by Ger et al. on a large monocentric population of 686 head and neck cancer patients, with the conclusion that radiomic features were not consistently associated with survival, neither in CT or PET images and even within patients undergoing the same imaging protocol [68].

Other articles focused on indirect prognosis factors such as human papillomavirus (HPV) positivity, as HPV-positive cancers have longer overall survival than HPV-negative ones [50]. In particular, Haider et al. tried to correlate radiomics findings with HPV-positivity in oropharyngeal squamous cell carcinoma: using 435 primary tumors (326 of which for training and the other 109 for validation), his model achieved an AUC of 0.83 [50].

One paper studied radiomics as a potential tool to assess node status: Chen et al. [39] used a model associating deep learning and radiomics to classify lymph nodes as normal, suspicious or involved, with a reported accuracy of 0.88.

For salivary gland cancers, radiomics was also studied for local control and overall survival, with good preliminary results [80,81,82]. However, the QS were low, ranging from 0 to 2.

### 3.7. Lung Cancer

PET radiomics in pulmonary oncology gathered 107 articles [83,84,85,86,87,88,89,90,91,92,93,94,95,96,97,98,99,100,101,102,103,104,105,106,107,108,109,110,111,112,113,114,115,116,117,118,119,120,121,122,123,124,125,126,127,128,129,130,131,132,133,134,135,136,137,138,139,140,141,142,143,144,145,146,147,148,149,150,151,152,153,154,155,156,157,158,159,160,161,162,163,164,165,166,167,168,169,170,171,172,173,174,175,176,177,178,179,180,181,182,183,184,185,186,187,188,189], all employing 18F-FDG. Only 8 studies included prospective cohorts (7.5%). The average number of patients included was 191.3 (range 30–1419) with 68/107 (63.6%) studies including more than 100 patients; 51/107 (47.7%) studies used dedicated validation cohorts. The main investigated topics were prognostic evaluation (50/107, 46.7%), benign versus malignant nodule characterization (13/107, 12.1%), epidermal growth factor receptor (EGFR) gene mutation prediction (12/107, 11.2%) and histological subtype prediction (9/107, 8%).

Among the most salient publications about tumor characterization, in a large study including 1419 patients (283 of whom in the testing set), Han et al. [131] developed a deep convolution neural network algorithm which achieved an accuracy of 0.841 for histologic subtype classification of non-small cell lung cancer. EGFR mutation detection, which conditions the use of dedicated targeted therapies, also benefitted from radiomics in a model developed by Chang et al. [90] on 583 patients (AUC: 0.84).

Radiomics was used for staging purposes by Zheng et al. [154], who used a radiomics-based model to predict mediastinal lymph node metastases in a population of 716 patients (501 of which included in the training cohort and the other 215 in the testing set): in the testing cohort, performances of the radiomics approach were significantly higher than clinical node staging (*p* = 0.037).

On the prognostic side, most studies focused on local control and treatment response prediction. Among them, a study on prospectively acquired data conducted by Mattonen et al. [88] (training: n = 145 patients; validation: n = 146 patients) was used to build a model that predicted recurrence/progression in non-small cell lung cancer (concordance of 0.74 in the testing set). A few studies have focused on the prediction of treatment side effects, especially radiation pneumonitis [93,139]. In the study conducted by Cui et al. [139], an externally validated deep learning model outperformed traditional normal tissue complication probability models in a multi-omics actuarial neural network architecture for prediction of radiation pneumonitis of grade 2 or higher.

### 3.8. Breast Cancer

We screened 32 publications on breast cancer [190,191,192,193,194,195,196,197,198,199,200,201,202,203,204,205,206,207,208,209,210,211,212,213,214,215,216,217,218,219,220,221], all employing 18F-FDG. The average number of enrolled patients was 126.8 (range 35–435), 18/32 (56.3%) studies including more than 100 patients; 3 studies (9.3%) were based on prospectively acquired data and 6/32 used an internal independent validation cohort (18.8%). Part of these studies was dedicated to investigating the correlation between texture parameters and immunohistochemical subtypes of breast cancer, in particular Liu et al. [210]. However, in a prospective study including 171 patients, Groheux et al. [202] did not find a high discriminative power for PET-derived texture metrics.

Other studies [190,214] have investigated the added value of texture parameters for predicting response to neoadjuvant chemotherapy and suggest a trend toward improved prediction models. In the largest study included for breast cancer, Lee et al. [214] concluded that the predictive power of a model incorporating both clinicopathological and texture factors was significantly higher than that of a model with clinicopathological factors only (AUC 0.80 vs. 0.73 *p* = 0.007).

### 3.9. Thyroid and Thymus

Six studies were available for thyroid tumors [222,223,224,225,226,227], all using 18F-FDG. The average number of patients was 89.0 (range 55–123); 3/6 (50.0%) studies included more than 100 patients, 1 was prospective and 1 used a validation cohort. Among them, 3/6 (50.0%) studies focused on 18F-FDG thyroid incidentalomas, 2 studies were interested in risk stratification in indeterminate thyroid nodules. The only study with a QS above 3, a prospective study of 123 patients conducted by de Koster et al. [225], reported no added value of radiomics for that purpose in 18F-FDG positive nodules.

Two retrospective studies were available for thymic epithelial tumors and 18F-FDG PET/CT [228,229], both including fewer than 50 patients and without validation cohorts.

## 4. Discussion

### 4.1. Quality Assessment and Textural Parameters Used

In this work, we considered 220 publications related to radiomics in supradiaphragmatic malignancies. Our composite score for the evaluation of the quality of the publications was low, estimated at 2.05/6, in good agreement with a previous work reporting low quality of radiomics publications [3]. Almost half of the publications had fewer than 100 patients, a number often cited as a threshold to avoid overfitting [230]. About 60% of the studies did not use an independent data set for model validation. This phenomenon, although explained by the difficulty of collecting data, limits the generalizability of the conclusions, as radiomics is dependent on acquisition protocols [2]. Although there are reserves about the quality of the work in previous publications, we observe an improvement over the years on our composite criterion combining the number of patients, the presence of a validation cohort, and the presence of prospective data. This improvement is probably due to the publication of guidelines and dedicated checklists to ensure proper methodology (e.g., the Image Biomarker Standardization Initiative [231] and the Radiomics Quality Score Checklist [232]).

In this review, most papers consider textural parameters up to second order. The consideration of higher order parameters is less frequently encountered (about 10%).

### 4.2. Trends and Topics

The number of studies on radiomics is exponentially increasing, relying on both machine learning and deep learning approaches. The most studied supradiaphragmatic cancers are, in order of frequency, lung cancer, head and neck cancers, and breast cancers. The majority of the studies here described focus on prognostic and treatment response objectives. 18F-FDG remains the most studied tracer, as expected due to its wide clinical use.

About brain tumors, PET radiomics and AI analysis could lead to a gain in more specific information on diagnosis and prognosis. Brain cancer patients usually have poor prognosis and new therapeutic strategies are needed to improve their outcomes [27]. PET radiomics and AI could help in the diagnosis of brain diseases with non-invasive methods and in the stratification of more aggressive histology at baseline, helping personalized and precision medicine in these conditions. Aminoacidic tracers [22,27] (e.g., 18F-DOPA, 11C-Methionine) and new targeted tracers could also increase the specificity of PET radiomics in certain diseases.

About lung tumors, PET radiomics and AI analysis have been widely evaluated in several settings. The number and the quality of available studies in the literature could help introducing these advanced systems in clinical practice. Probably, new studies should be focused on external validation cohorts to clearly assess the clinical usefulness of PET radiomics and AI in lung cancer, such as in distinguishing lung metastases and primary tumors with different histology and prognosis.

Similarly, for head and neck tumors, PET radiomics and AI analysis should spread in a clinical routine evaluation to definitively allow personalized and precision medicine for these patients. Some limitations emerged in 18F-FDG PET/CT applications in head and neck tumors for not being able to distinguish between inflammatory activation in some tissues and the localization of the tumor [47]. PET radiomics and AI analysis could help physicians to overcome these limitations.

In breast cancer, PET/CT has a great value for staging and restaging purposes [191]. The limited avidity of 18F-FDG in some primary breast tumors could be a limitation in the application of PET radiomics and AI analysis. Nevertheless, new tracers such as 18F-FAPI and monoclonal antibodies could be used in the near future to study the textural heterogeneity in primary breast tumors, thanks to PET radiomics and AI analysis.

18F-FDG PET/CT still has limited indications in thyroid cancer [226], mainly for restaging of differentiated carcinomas and staging of anaplastic carcinomas. Therefore, PET radiomics and AI analysis should be further evaluated.

Given the increasing number of immunotherapies in metastatic cancer from different primary tumors, PET radiomics and AI analysis may be considered to better evaluate cases of stable disease or pseudo-progression due to inflammatory reaction to immunomodulators, such as in lung cancer or breast cancer.

### 4.3. Limitations

Our review has a certain number of limitations: first, we set an arbitrary threshold of 30 patients to eliminate studies that were too exposed to an overfitting bias. One of the disadvantages of this selection is the potential elimination of rare pathologies from this review, as previously reported [233].

The scale used to assess the quality of the articles was practical but rather simplistic. This score made it possible to evaluate a large number of articles with a fairly high reproducibility (11 discrepancies between the reading sessions) at the expense of a thorough analysis of the methods.

Finally, and because the textural parameters and methods used are very different from one article to another, even for similar subjects or cancers it was challenging to aggregate and compare the articles between them.

## 5. Conclusions

Radiomics and AI are upcoming fields in nuclear oncology, especially in brain, head and neck, thyroid, and breast cancers. Although technically demanding, new contributions with robust validation cohorts and guidelines for clinical practice applications will surely continue helping to improve the quality and the impact of the reported studies over the years.

## Figures and Tables

**Table 1:** Inclusion and exclusion criteria (PICOS systematization).

Parameter	Inclusion Criteria	Exclusion Criteria
Patients	Patients with any supradiaphragmatic cancer	Patients with a not strictly supradiaphragmatic malignancy
Intervention	Radiomics analysis on PET studies	
Comparator	Diagnostic performances	
Outcome	Primary outcome measures, diagnostic accuracy, area under curve	
Study design	Any trials, retrospective, prospective or concurrent cohort studies. At least 30 patients. Published in English.	Reviews, expert opinions, comments, letters to editor, case reports, studies on animals or phantoms, conference reports. Fewer than 30 patients. Studies with no outcomes reported. Published in any language other than English

**Table 2:** Mean quality scores and number of publications per year on PET(/CT) radiomics.

Year	Quality Score (Mean-95%CI)	Number of Publications
2013	1 [-]	2
2014	0.80 [0;2.44]	5
2015	1.33 [0;4.28]	6
2016	1.86 [0;4.49]	7
2017	1.72 [0;4.48]	18
2018	1.96 [0;4.71]	28
2019	1.88 [0;4.29]	42
2020	2.31 [0.34;4.27]	50
2021	2.33 [0.11;4.55]	52
2022 *	2.40 [0.29;4.51] *	10 *

* partial data.

## Data Availability

The datasets generated during the current study are available from the corresponding author on reasonable request.

## Supplementary Materials

The following are available online at https://www.mdpi.com/article/10.3390/diagnostics12061329/s1, Table S1: A study characteristics table.
